# Netupitant versus aprepitant: model-predicted neurokinin-1 receptor occupancy and implications for long-delayed nausea and vomiting prevention

**DOI:** 10.1007/s00520-026-11175-y

**Published:** 2026-09-05

**Authors:** Hirotoshi Iihara, Giampaolo Bianchini, Matti Aapro, Luca Licata, Alberto Bernareggi, Rudolph M. Navari, Yeon Hee Park, Karin Jordan, María Vidal, Florian Scotté, Eric J. Roeland, Lee Schwartzberg, Hope S. Rugo

**Affiliations:** 1 Department of Pharmacy, Gifu University Hospital, Gifu, Japan; 2 Department of Medical Oncology, IRCCS San Raffaele Hospital, Milan, Italy; 3 School of Medicine and Surgery, Vita-Salute San Raffaele University, Milan, Italy; 4 Genolier Cancer Center, Clinique de Genolier, Genolier, Switzerland; 5 Clinical Pharmacology Consultant, Concorezzo, Italy; 6 World Health Organization, Mount Olive, AL USA; 7 Division of Hematology-Oncology, Department of Medicine, Samsung Medical Center, Sungkyunkwan University School of Medicine, Seoul, Republic of Korea; 8 Department of Hematology, Oncology and Palliative Medicine, Ernst Von Bergmann Hospital, Potsdam, Germany; 9 Department of Medicine V, Hematology, Oncology and Rheumatology, University of Heidelberg, Heidelberg, Germany; 10 Department of Medical Oncology, Hospital Clinic; Translational Genomics and Targeted Therapies in Solid Tumors, IDIBAPS; Department of Medicine, University of Barcelona, Barcelona, Spain; 11 Interdisciplinary Cancer Course Department, Gustave Roussy Cancer Institute, Villejuif, France; 12 Oregon Health and Science University, Knight Cancer Institute, Portland, OR USA; 13 Renown Health-Pennington Cancer Institute, University of Nevada, Reno, NV USA; 14 Department of Medical Oncology & Therapeutics Research, City of Hope Comprehensive Cancer Center, Duarte, CA USA; 15 Cancer Center, Gifu University Hospital, Gifu, Japan

**Keywords:** Nausea, Vomiting, Antibody–drug conjugate, CINV, Netupitant, Aprepitant

## Abstract

**Purpose:**

Nausea and vomiting beyond 5 days after emetogenic chemotherapy or antibody–drug conjugate (ADC) therapy are common, yet the role of neurokinin-1 (NK_1_) receptor antagonists in this setting remains underrecognized. We used pharmacokinetic/pharmacodynamic (PK/PD) modeling to estimate the NK_1_ receptor occupancy (RO), a proxy for clinical efficacy, for up to 20 days after a single 300 mg dose of oral netupitant, 3-day oral aprepitant (125 mg on day 1; 80 mg on days 2–3), or a single 165 mg dose of oral aprepitant.

**Methods:**

Data from previous PK studies were analyzed by compartmental modeling. Positron emission tomography studies assessing striatal NK_1_ RO were used to develop maximum drug effect PD models, which were fitted to NK_1_ RO data as a function of plasma concentrations.

**Results:**

Model predicted NK_1_ RO exceeded 90% at 3 h for all treatments. Thereafter, RO declined more gradually with netupitant (76%, 70%, 60%, and 21% on days 5, 7, 10, and 20, respectively) than with 3-day aprepitant (81%, 39%, 3%, and negligible) or single-dose aprepitant (45%, 10%, < 1%, and negligible). The half-life of netupitant was ~ 6.1 times longer than aprepitant’s. Netupitant plasma concentration remained above the effective concentration for 50% NK_1_ RO (EC_50_) through day 10, whereas aprepitant concentrations fell below the EC_50_ by ~ days 7 and 5 after repeated and single dosing, respectively.

**Conclusions:**

Single-dose netupitant maintained NK_1_ RO substantially longer than repeated- and single-dose aprepitant, suggesting greater potential for prolonged prevention of nausea and vomiting in ADC-treated patients. Prospective clinical validation of these model predictions would be beneficial.

**Supplementary Information:**

The online version contains supplementary material available at https://doi.org/10.1007/s00520-026-11175-y.

## Introduction

Chemotherapy-induced nausea and vomiting (CINV) remains a significant challenge in oncology, negatively impacting patient quality of life, treatment adherence, and clinical outcomes [1–4]. While acute (0–24 h) and delayed (> 24–120 h) CINV are well-characterized, there is growing recognition of “long-delayed” CINV extending beyond 120 h [5, 6]. Real-world evidence suggests symptoms can persist 8–14 days post-chemotherapy, affecting a substantial portion of patients receiving highly or moderately emetogenic regimens [7].

The clinical relevance of long-delayed CINV is further heightened by the increasing use of antibody–drug conjugates (ADCs) [6, 8–10]. Agents such as trastuzumab deruxtecan, datopotamab deruxtecan, and sacituzumab govitecan are associated with nausea and vomiting that can persist for 10 days or longer, potentially leading to dose reductions and compromised efficacy [6, 9, 11]. This is particularly relevant because ADCs are often administered over multiple cycles and extended treatment durations, potentially exposing patients to recurrent episodes of prolonged nausea and vomiting. These trends underscore the urgent need for antiemetic protection that extends well beyond the standard 5-day window.

Neurokinin-1 (NK_1_) receptor antagonists are critical for managing delayed emesis. The fixed-combination NEPA (netupitant/palonosetron) utilizes oral or intravenous (IV) (fos)netupitant, an NK_1 _receptor antagonist with a significantly longer half-life than oral or IV (fos)aprepitant [12–14]. Pharmacokinetic/pharmacodynamic (PK/PD) modeling suggests that NK_1_ receptor occupancy (RO) serves as a proxy for clinical efficacy. Previous positron emission tomography (PET)-based studies have demonstrated a relationship between NK_1 _RO and antiemetic efficacy, supporting the use of RO as a pharmacodynamic surrogate. While historical data for aprepitant suggest a requirement for > 90% RO for optimal control [15, 16], clinical studies suggest that netupitant may be efficacious at a lower threshold, potentially allowing for a more prolonged duration of action. Indeed, the 100 mg lowest dose of netupitant demonstrated similar efficacy to the 300 mg standard dose, despite the lower NK_1_RO [17].

While there is strong pharmacological rationale for prolonged RO, clinical data directly comparing NK_1_ receptor antagonists for long-delayed CINV beyond day 7 remain limited. In this context, PK/PD modeling may provide insight into the differences in the duration of pharmacologic activity among NK_1_ receptor antagonists. The objective of this analysis was to estimate the time course of NK_1_ RO for up to 20 days following a single 300 mg oral dose of netupitant and compare it with standard aprepitant regimens.

## Methods

### Source datasets and ethics

Pharmacokinetic (PK) and pharmacodynamic (PD) data used for modeling were obtained from previously published human studies [15, 18–20]. All source studies were approved by local independent ethics committees or institutional review boards and conducted in compliance with Good Clinical Practice guidelines and the Declaration of Helsinki. Subject demographics (age and sex) were comparable across the netupitant and aprepitant PK and PD studies.

- *Netupitant data*: The mean netupitant plasma concentration–time profile analyzed by compartmental modeling was obtained from a Phase I PK study in 24 healthy adults receiving a single oral dose of NEPA (netupitant 300 mg/palonosetron 0.5 mg) in fasted state [20]. In vivo NK_1_ RO% data were derived from a PET study in 6 healthy volunteers utilizing the tracer [^11^C]‐GR205171 following oral netupitant administration [18].
- *Aprepitant data*: Mean aprepitant plasma concentrations were sourced from a PK study after a 125 mg oral dose of aprepitant in 25 healthy subjects in fasted state [19]. Baseline and trough NK_1_ RO values were obtained from two single-blind, placebo-controlled PET studies involving 16 healthy volunteers using the tracer [^18^F]SPA-RQC after 14 consecutive days of dosing [15].

The striatum was selected as the target region of interest for all dynamic PET analyses because it contains the highest human density of NK_1 _receptors and provides the largest specific PET signal [21]. Details on source data can be found in the Supplementary Material.

### Pharmacometric modeling approach

- *PK modeling*: Best-fit of the mean plasma concentration–time curves of netupitant and aprepitant were performed using the Microsoft 365 Excel PKSolver Add-in. A two-compartment open model for oral administration with first-order input with lag-time and a one-compartment open model for oral administration with first-order input with no lag-time were fitted to netupitant and aprepitant curves, respectively. Primary and secondary PK parameter definitions, along with the foundational model differential equations, are provided in the Supplementary Material.
- *PD modeling*: For netupitant, parameters were taken from a previously established sigmoid maximum drug effect (E_max_) model relating striatal NK_1 _RO% to plasma concentrations [18] where E_max_ is the maximal occupancy of NK_1_ receptors (NK_1_ RO%), Conc is the plasma concentration of netupitant at the PET scans, EC_50_ is the plasma concentration at which 50% of E_max_ is achieved, and γ is a slope parameter reflecting the shape of the curve. The software used was WinNonlin Professional Edition Version 4.1.b (Pharsight Corporation, Mountain View, CA, USA). For aprepitant, PD model parameters were estimated by fitting a simple E_max_ model (*γ* = 1) to the NK_1 _RO% data in the striatum after administration as a function of aprepitant plasma concentrations [15] using the software Microsoft 365 Excel PKSolver Add-in. The E_max_model parameters reported in the publication [15] appeared to lack sufficient accuracy. Therefore, they were recalculated to allow an appropriate PK/PD correlation.

PD parameter definitions and E_max_ model equations are provided in the Supplementary Material.

### Simulation of NK_1_ RO after netupitant and aprepitant administration

Using the PD model-estimated parameters and the PK model-predicted plasma concentrations of netupitant and aprepitant at any time, the time course of striatal NK_1_ RO was simulated across a 20-day (480-h) time period.

Occupancy profiles were simulated for three distinct comparator antiemetic regimens:

1. A single oral dose of *netupitant 300 mg* (the oral dose bioequivalent to intravenous [IV] fosnetupitant 235 mg).
2. A single oral dose of *aprepitant 165 mg* (the oral dose bioequivalent to IV fosaprepitant 150 mg).
3. A standard 3-day oral clinical regimen of *aprepitant* (125 mg on day 1, followed by 80 mg on Days 2 and 3).

Comparative evaluation points were targeted at Days 5, 7, 10, and 20. Day 5 corresponds to the traditional end of CINV assessment, whereas Days 7, 10, and 20 were selected to evaluate extended RO relevant to long-delayed CINV and ADC-associated nausea and vomiting.

## Results

### Pharmacokinetic modeling

**Fig. 1:**
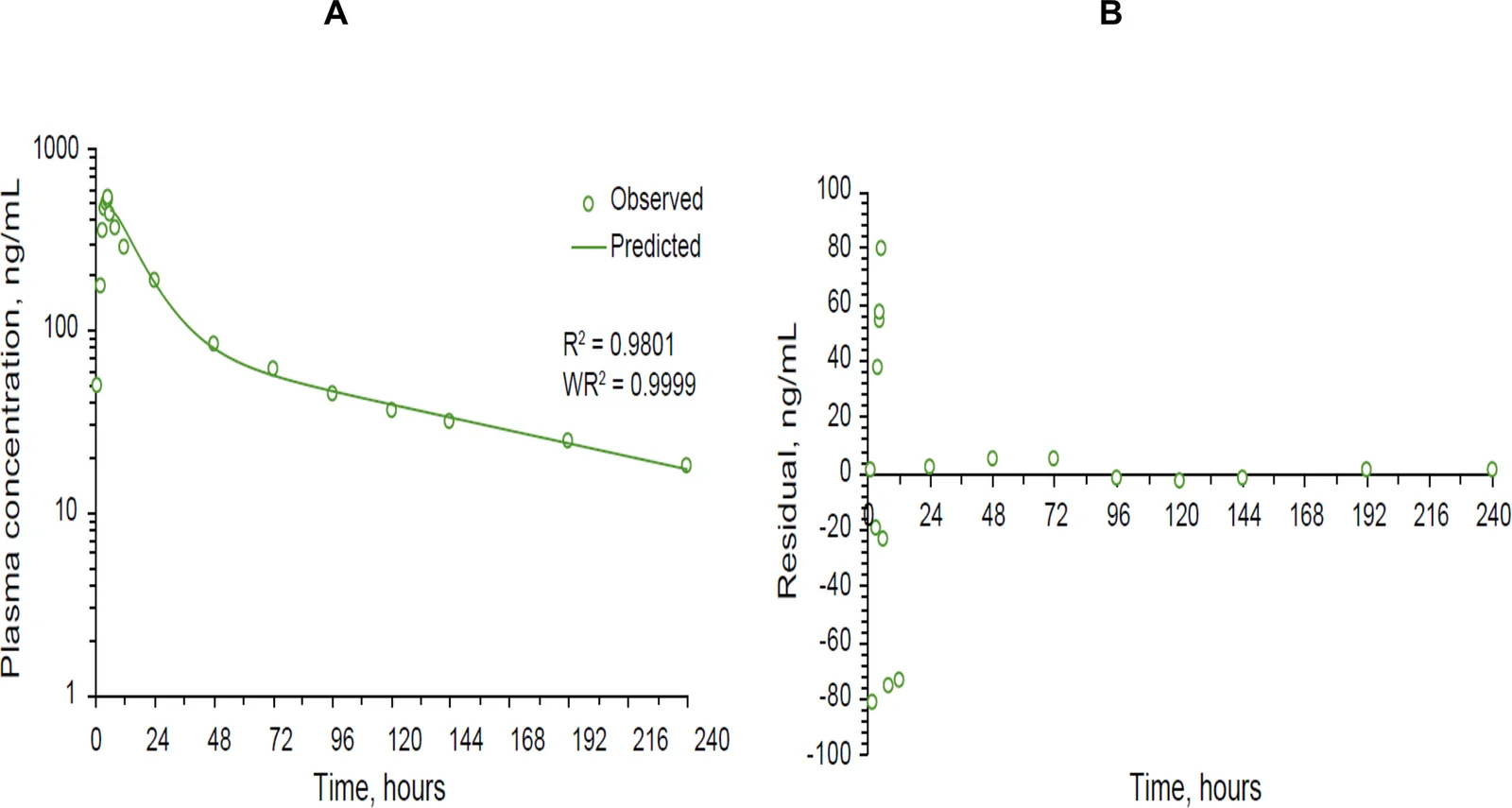
Best fit curve of netupitant mean plasma concentrations as a function of time (**A**) and plot of residuals (**B**). *R*^2^ is the determination coefficient; *WR*^2^ is the weighted determination coefficient

**Fig. 2:**
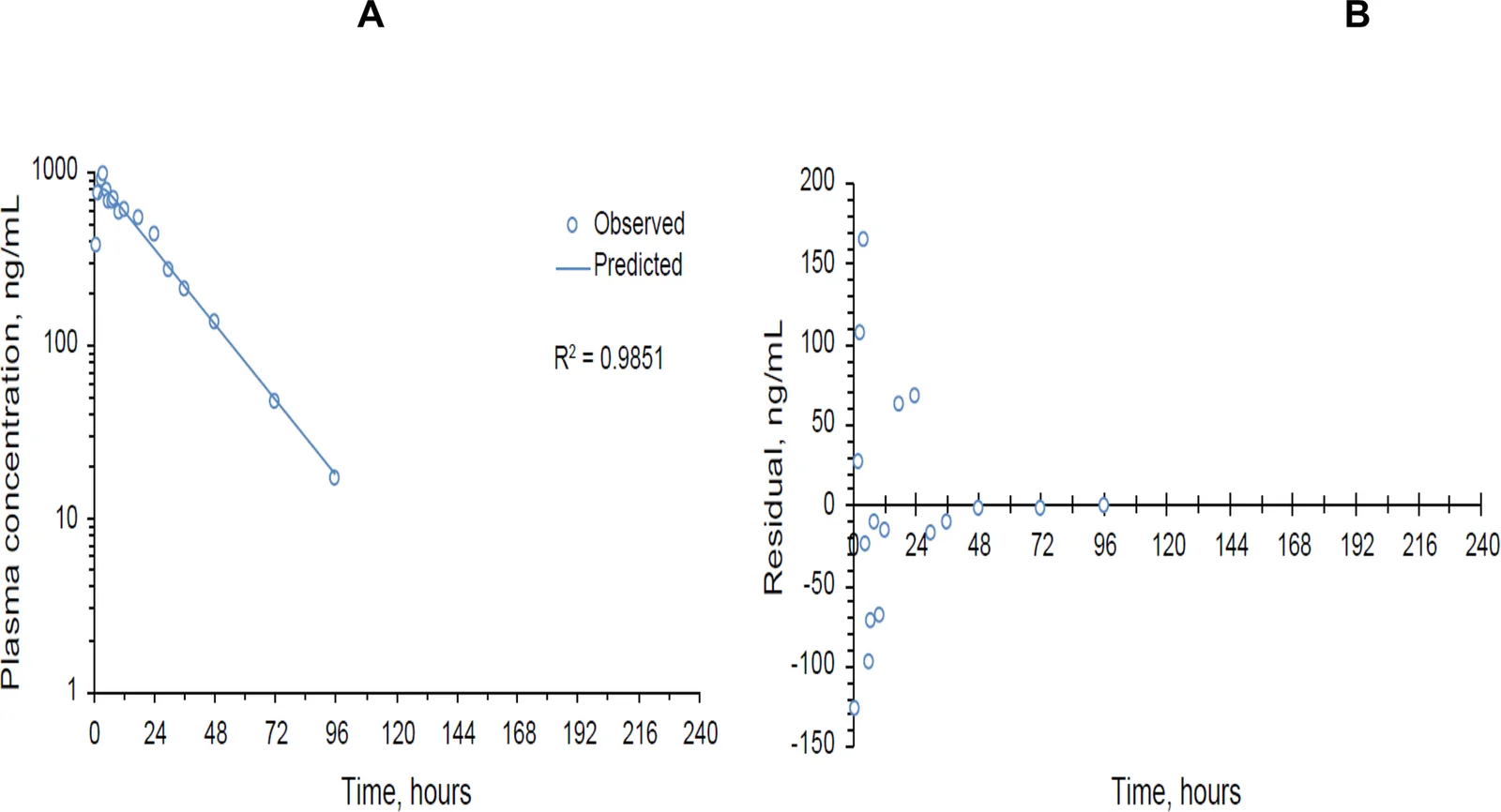
Best fit curve of aprepitant mean plasma concentrations as a function of time (**A**) and plot of residuals (**B**)

Compartmental PK analysis characterized the distinct plasma concentration–time profiles of netupitant and aprepitant. Estimated primary and secondary PK parameters are provided in Table 1. Model diagnostic parameters demonstrated excellent goodness-of-fit for both agents (WR^2^ = 0.9999 for netupitant; *R*^2^ = 0.9851 for aprepitant). Figures 1 and 2 illustrate the best fit curve on semi-logarithmic scale (A) and the plot of residuals (B) for netupitant and aprepitant, respectively.

**Table 1:** Primary and secondary PK parameters for netupitant and aprepitant from compartmental PK modeling

Parameter	Unit	Netupitant	Aprepitant
Dose	mg	300	125
A	ng/mL	678.3	996.0
B	ng/mL	89.4	-
C	ng/mL	767.7	-
K_a_	h^−1^	0.45357	0.79217
α	h^−1^	0.07964	-
β	h^−1^	0.00682	0.04165
K_10_	h^−1^	0.03248	0.04165
K_12_	h^−1^	0.03726	-
K_21_	h^−1^	0.01673	-
t_lag_	h	0.825	-
t_1/2,Ka_	h	1.53	0.88
t_1/2,α_	h	8.70	-
t_1/2,K10_	h	21.3	16.6
t_1/2,β_	h	101.6	16.6
C_max_	ng/mL	462.4	801.3
C_max_/D	ng/mL/mg	1.54	6.41
t_max_	h	5.77	3.92
AUC_0-inf_	ng•h/mL	19,929.9	22,654.0
AUC_0-inf_/D	ng•h/mL/mg	66.43	181.23
CL/F	L/h	15.05	5.52
CL_2_/F	L/h	17.27	-
V/F	L	463.5	132.5
V_2_/F	L	1032.3	-

Where: *A*, *B*, and *C* are macroconstants; *K*_a_ is the absorption rate constant; *α* is the distribution rate constant; *β* is the terminal rate constant; *K*_10_ is the elimination rate constant from the central compartment; *K*_12_ and *K*_21_ are the transfer rate constants from the central compartment 1 to the peripheral compartment 2, and vice versa; *t*_lag_ is the lag-time; *t*_1/2,Ka_, *t*_1/2,α_, and *t*_1/2,K10_ are the half-lives associated to the absorption, distribution, and elimination phases, respectively; *t*_1/2,β_ is the half-life of the terminal phase, *C*_max_ is the maximum model-predicted concentration; *t*_max_ is the model-predicted peak time; *AUC*_0-inf_ is the model-predicted area under the plasma concentration–time curve from time zero to infinity; *AUC*_0-inf_/D is the *AUC*_0-inf_ normalized for the administered dose (D); *CL*/*F* is the estimated systemic clearance over the bioavailability (F); *CL*_2_/*F* is the inter-compartmental clearance over F; *V*/*F* and *V*_2_/*F* are the volume of distribution over F of central and peripheral compartments, respectively

#### Netupitant

Compartmental PK modeling of the netupitant mean plasma concentration–time curve revealed a gastrointestinal absorption lag (t_lag_) of 0.825 h, an absorption rate constant (K_a_) of 0.45357 h^−1^, an absorption half-life (t_1/2,Ka_) of 1.53 h, and a peak concentration (C_max_) of 462.4 ng/mL occurring near 6 h post-dose. Netupitant exhibited a wide volume of distribution in the central (V/F = 463.5 L) and peripheral (V_2_/F = 1032.3 L) compartments (total volume of distribution: 1495.8 L). The inter-compartmental transfer rate constants K_12_ and K_21_ were low, particularly the rate of netupitant return from the peripheral to the central compartment, K_21_. Netupitant is slowly eliminated from the body, consistent with its estimated low clearance value (CL/F) of 15.05 L/h and long terminal phase half-life (t_1/2,β_) of 101.6 h.

#### Aprepitant

Compartmental PK modeling of the aprepitant mean plasma concentration–time curve yielded a C_max_ of 801.3 ng/mL at approximately 4 h post-dose, with an absorption profile comparable to that of netupitant, as shown by comparable t_max_, K_a_ and t_1/2,Ka_ values **(**Table 1**)**. However, as compared to netupitant, aprepitant displayed a lower volume of distribution (V/F = 132.5 L) and a substantially shorter terminal half-life (t_1/2,__β_) of 16.6 h, despite its lower systemic clearance (CL/F = 5.52 L/h).

**Fig. 3:**
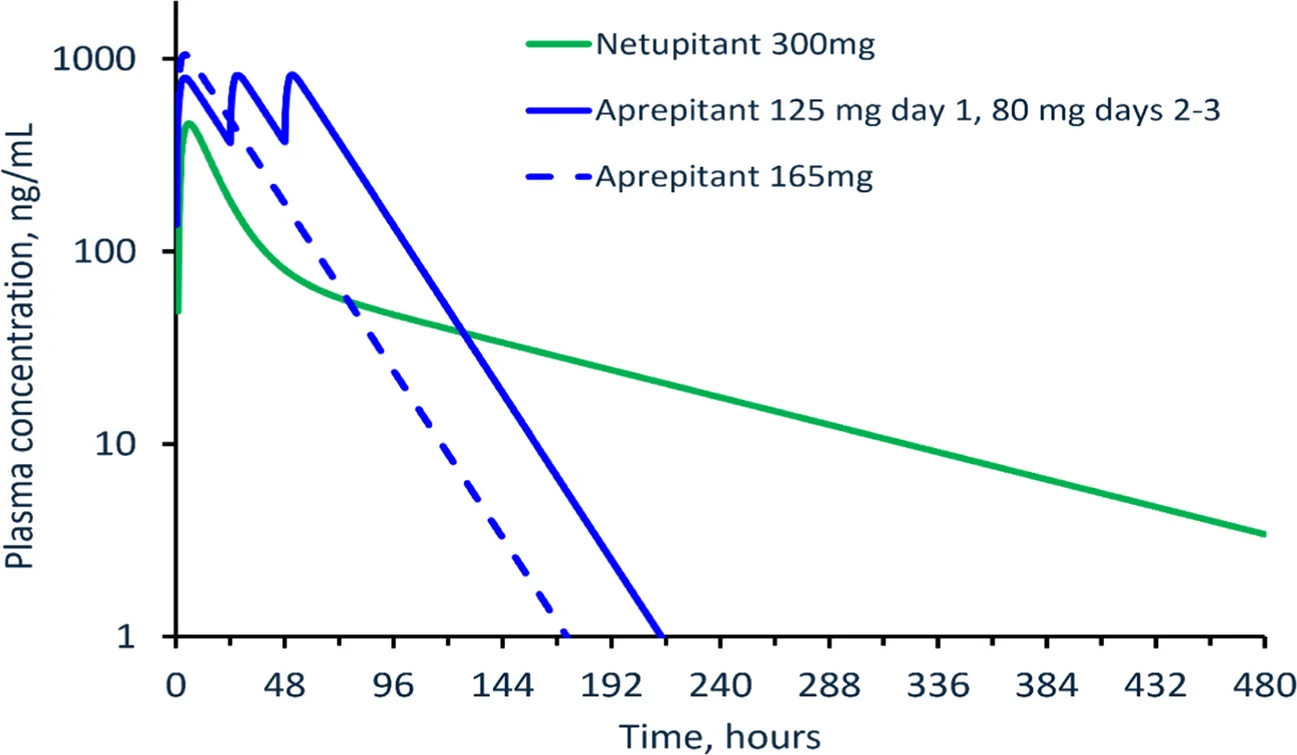
PK-model-predicted plasma concentrations of netupitant and aprepitant over time

Model-predicted plasma concentration–time profiles over the 20-day post-administration period after netupitant administration of a single oral dose of 300 mg, and aprepitant administration of a single oral dose of 165 mg as well as multiple dosing with 125 mg on day 1 and 80 mg on days 2–3, assuming PK linearity in the tested dose range, are illustrated in Fig. 3. These plasma concentration–time profiles show that netupitant concentrations decline more slowly and exceed those of aprepitant beyond approximately 144 h after dosing due to its persistent terminal half-life.

### Pharmacodynamic modeling

The pharmacodynamic (PD) parameters used to generate the pharmacokinetic/pharmacodynamic (PK/PD) correlations are detailed in Supplementary Table 1.

#### Netupitant

Fitting the sigmoid E_max_model to PET data [18] yielded an E_max_ of 93.0% and an EC_50_ of 10.2 ng/mL and *γ* = 1.1. Netupitant plasma concentrations remained above this EC_50_ threshold beyond 240 h (day 10).

#### Aprepitant

Simple E_max_modeling of aprepitant PET data [15] achieved high accuracy (*R*^2^ = 0.9958), defining an E_max_ of 98.1% and an EC_50_ of 10.4 ng/mL. In contrast to netupitant, aprepitant plasma concentrations dropped sharply below its EC_50_ by 120 h following a single 165 mg dose, and by 168 h following the 3-day regimen.

Although the EC_50_ for NK_1_ RO was similar for netupitant and aprepitant (10.2 ng/mL and 10.4 ng/mL, respectively), the much longer terminal half-life of netupitant compared with aprepitant caused netupitant concentration to remain substantially above the EC_50_ at day 10, while aprepitant concentration was an order of magnitude lower than the EC_50_ at day 10 (0.34 ng/mL for the 3-day aprepitant course and 0.06 ng/mL for aprepitant 165 mg) (Supplementary Table 1).

### Predicted time course of NK_1_ RO

**Fig. 4:**
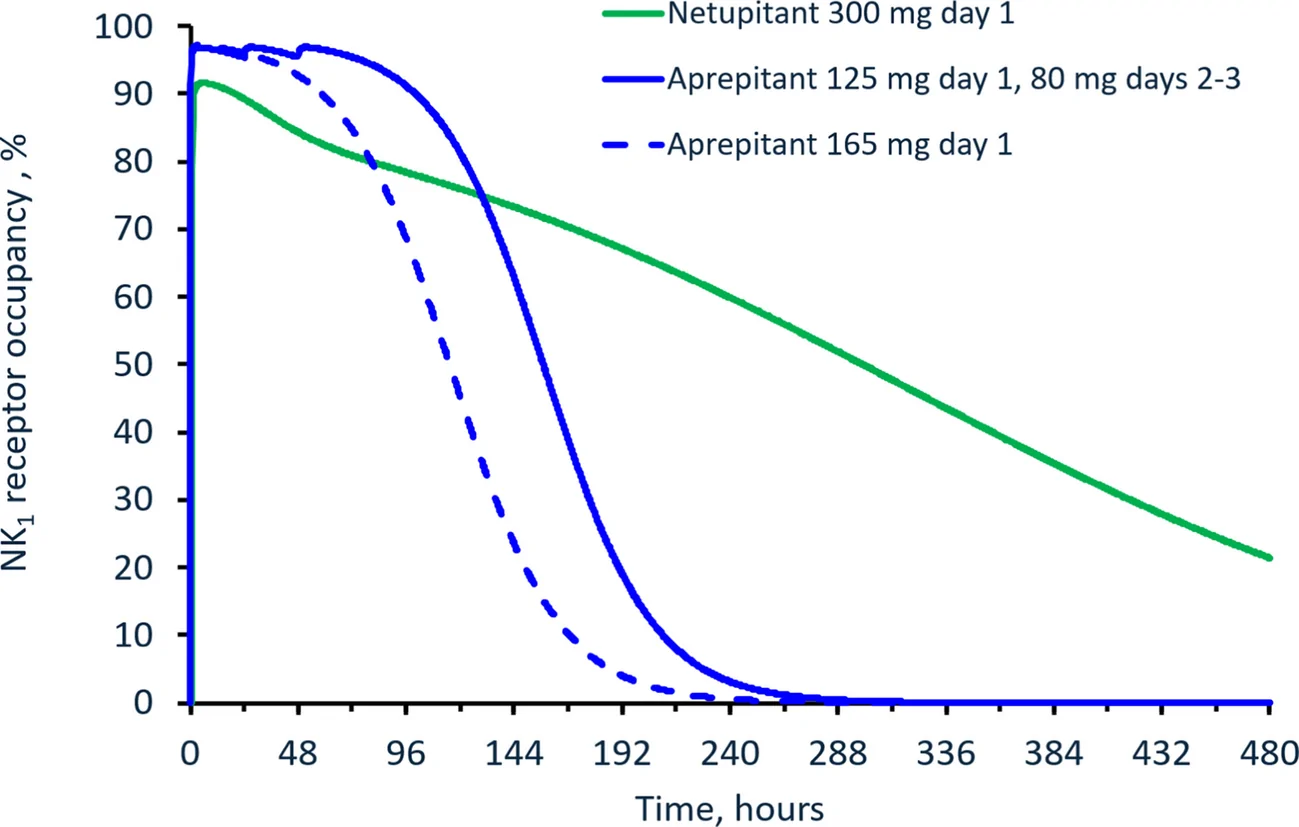
Model-predicted NK_1_ receptor occupancy in the striatum as a function of time after netupitant and aprepitant oral administration

Model-predicted NK_1_ RO in the striatum as a function of time after netupitant and aprepitant oral administration is depicted in Fig. 4. All three evaluated antiemetic regimens achieved robust initial target engagement, exceeding 90% RO within 3 h of dosing on day 1. Up to day 5 (120 h), target occupancy remained high for both single-dose netupitant 300 mg (RO% = 76% at 120 h) and the 3-day oral aprepitant regimen (RO% = 81% at 120 h), while single-dose aprepitant 165 mg fell to 45% at 120 h. Following day 5, striatal engagement diverged significantly. Receptor occupancy declined gradually for netupitant but rapidly for aprepitant. At day 7 (168 h) predicted RO was 70% for netupitant compared with 39% for the 3-day aprepitant regimen and 10% for the single 165 mg aprepitant dose. At day 10 (240 h), RO was sustained at 60% for netupitant but declined to 3% and < 1% for the 3-day and single-dose aprepitant regimens, respectively. On day 20 (480 h) netupitant still retained 21% RO, whereas occupancy with aprepitant was negligible for both comparator regimens.

## Discussion

The management of long-delayed chemotherapy-induced nausea and vomiting (CINV) remains an underrecognized unmet need, particularly with the emergence of antibody–drug conjugates (ADCs) like trastuzumab deruxtecan and sacituzumab govitecan. These agents often exhibit late-onset symptoms that persist 10 to 20 days post-administration, coinciding with the end of a treatment cycle. Because experiencing CINV on days 4 and 5 is a primary risk factor for further long-delayed symptoms, providing antiemetic coverage with a long duration of action is critical. Our PK/PD modeling supports the potential of netupitant (as oral NEPA or IV fosnetupitant) to provide this extended protection. PK/PD modeling provides a useful framework for addressing clinically relevant questions that have not yet been evaluated directly in prospective trials, particularly in settings where long-delayed efficacy data remain limited.

### Mechanistic basis for sustained occupancy

Our analysis indicates that netupitant maintains higher plasma concentrations and superior striatal NK_1_ RO% compared to aprepitant beyond day 5. This sustained pharmacological activity is largely driven by netupitant’s distinct pharmacokinetic profile, characterized by a significantly longer terminal half-life (101.6 h vs. 16.6 h) and a larger volume of distribution (V/F) compared to aprepitant.

The larger volume of distribution of netupitant was expected, given its higher lipophilicity (estimated partition coefficient, logP, of 5.48 for netupitant vs 2.44 for aprepitant) [22, 23]. In preclinical studies, this broader distribution is also associated with greater brain permeability for netupitant compared with aprepitant. Specifically, the estimated brain-to-plasma ratio for netupitant in rats ranges from 2.4 to 4.9 after a single IV dose, and from 0.8 to 2.0 at steady state in a 4-week oral toxicology study [data on file]. In contrast, the brain-to-plasma ratio for aprepitant in ferrets at steady state ranges from 0.4 to 0.6 [24]. This indicates that the brain-to-plasma ratio for netupitant is 1.3- to 12-fold higher than that of aprepitant. The greater overall V/F value of netupitant may explain its lower values of dose normalized C_max_ (1.54 vs 6.41 ng/mL/mg) and AUC_0-inf_ (66.43 vs 181.23 ng•h/mL/mg), as well as its longer t_1/2,β_ (101.6 vs 16.6 h), as compared with aprepitant, despite the almost threefold lower aprepitant clearance (CL/F: 5.52 L/h vs 15.05 L/h) value (Table 1). These PK properties clearly favor the sustained and prolonged NK_1_ RO% properties of netupitant.

### Clinical relevance of occupancy thresholds

Because NK_1_ RO has been shown to correlate with antiemetic activity in PET-based pharmacodynamic studies, RO provides a clinically relevant surrogate for comparing the duration of pharmacologic activity among NK_1 _receptor antagonists. A key finding of this study is the prolonged duration that netupitant remains above its effective activity threshold. Previous independent studies suggest that while aprepitant may require >90% RO% for maximal efficacy [15], netupitant may provide effective control at lower occupancy levels [17, 18, 25]. On day 4 after NEPA (300 mg netupitant, 0.5 mg palonosetron) administration, 98% of patients receiving highly emetogenic chemotherapy had a complete response (no emesis and no rescue medication) [17], a time point that corresponds with an estimated NK_1 _receptor occupancy in the striatum of 78% [25]. Moreover, since the 100-mg dose of netupitant can effectively control nausea and vomiting over the 5-day period following cisplatin-based chemotherapy—with an estimated NK_1 _receptor occupancy in the striatum on day 4 close to 50% at this dose [18]—it may be assumed that 50% occupancy is the threshold for effective antiemetic activity for netupitant, which is substantially lower than the 90% threshold required for aprepitant maximal efficacy [15].

Our model predicts that single-dose netupitant maintains RO% levels of 70% and 60% on days 7 and 10, respectively, remaining above even conservative thresholds. In contrast, aprepitant RO% falls to negligible levels (< 3%) by day 10, regardless of the regimen used.

Previous PK/PD modeling has also demonstrated broadly similar prolonged NK_1 _RO with both rolapitant and netupitant [26], illustrating that plasma pharmacokinetic characteristics alone do not fully predict the duration of central NK_1_ receptor blockade. These observations further support the value of RO–based PK/PD modeling for comparing NK_1_ receptor antagonists beyond conventional plasma pharmacokinetic measures.

### Therapeutic implications

PK/PD modeling predicts that netupitant and aprepitant provide comparable RO during the first days following administration, when occupancy levels remain above their respective activity thresholds. Differences emerge primarily beyond day 4, when RO declines rapidly for aprepitant but remains sustained for netupitant. These modeling results are consistent with clinical data showing the superiority of NEPA over aprepitant in preventing nausea and vomiting beyond the first 120 h [27, 28]. For patients receiving ADCs or highly emetogenic chemotherapy, the predicted maintenance of NK_1 _RO for at least 10 days suggests that netupitant may provide more comprehensive coverage throughout the treatment cycle. These modeling findings are promising. They should be interpreted as supporting prolonged pharmacologic activity rather than direct clinical superiority in CINV prevention. Importantly, these predictions are consistent with available clinical evidence. Pooled analyses of randomized studies, an individual-patient-data meta-analysis, and prospective comparative studies have demonstrated improved control of nausea and vomiting with NEPA relative to aprepitant-based regimens during the later days of the assessment period [29, 30]. Similarly, an exploratory analysis of a phase 3 study showed superior protection through day 7 with fosnetupitant compared with fosaprepitant [27]. Together, these findings provide clinical support for the prolonged RO predicted by the present model. Because the IV prodrugs fosnetupitant and fosaprepitant are bioequivalent to the oral doses modeled here, these conclusions likely extend to IV administration as well.

In summary, the PK/PD properties of netupitant, including in particular its long half-life and wide volume of distribution, facilitate sustained RO that meets the clinical requirements for managing long-delayed CINV. This pharmacological profile offers a robust rationale for its use with modern oncology regimens where extended symptom control is of paramount importance.

### Limitations

This study has some limitations. First, it was a retrospective PK/PD modeling analysis rather than a prospective clinical study. Second, the models were developed using mean PK data and PET RO data derived from independent studies involving relatively small numbers of healthy volunteers, employing different study designs and assessment methodologies. In particular, NK_1_ RO was determined using different PET tracers, which may introduce variability and limit direct comparability across studies.

The analysis also assumes that the PK and PD relationships observed in healthy volunteers are applicable to patients with cancer. Although no clinically meaningful pharmacokinetic differences have been reported between healthy subjects and patients with cancer for either netupitant or aprepitant [12, 31], patient-specific risk factors for CINV may influence clinical outcomes and are not captured by the current model.

In addition, the original studies evaluated the antiemetic agents in isolation and did not assess potential interactions with concomitant anticancer therapies. As with any modeling exercise, the predictions are dependent on the quality and assumptions of the source datasets and should therefore be interpreted cautiously.

Despite these limitations, PK/PD modeling provides a useful approach for evaluating questions that have not yet been addressed in prospective clinical trials. The findings should be considered hypothesis-generating and warrant prospective clinical validation, particularly in patients receiving ADCs and other therapies associated with long-delayed nausea and vomiting.

## Conclusion

A single 300-mg oral netupitant administration maintained NK_1_ RO much longer than a single 165 mg or the standard 3-day aprepitant regimen (125 mg on day 1 and 80 mg on days 2–3). Based on these PK/PD model predictions and considering NK_1_ RO as a pharmacodynamic surrogate of antiemetic efficacy, netupitant may provide more prolonged pharmacologic coverage than aprepitant, potentially translating into improved protection against long-delayed nausea and vomiting. These findings may be particularly relevant for patients receiving ADCs such as trastuzumab deruxtecan and sacituzumab govitecan, as well as conventional chemotherapy with prolonged emetic risk. Antiemetic prophylaxis with NEPA may help provide sustained control of nausea and vomiting over an extended period, supporting treatment adherence, quality of life, and optimal continuous anticancer therapy.

## Supplementary Information

Below is the link to the electronic supplementary material.

ESM 1 (DOCX 68.2 KB)

## Data Availability

Individual patient-level data will not be shared.

## Declarations

### Disclaimer

The authors are fully responsible for all content and editorial decisions for this manuscript.
