# Supplementary material for: Netupitant versus aprepitant: model-predicted neurokinin-1 receptor occupancy and implications for long-delayed nausea and vomiting prevention
Source: Support Care Cancer. 2026 Sep 5;34(10):933. doi: 10.1007/s00520-026-11175-y (PMC13546154; doi:10.1007/s00520-026-11175-y)
Supplement: Supplementary file 1 [file 520_2026_11175_MOESM1_ESM.docx]

**Supplementary Material**

Supplement to: Iihara H et al. Netupitant Versus Aprepitant: Model-Predicted Neurokinin-1 Receptor Occupancy and Implications for Long-Delayed Nausea and Vomiting Prevention

**Detailed METHODS**

Data for PK/PD modeling were taken from previous studies in humans, which are reported in full elsewhere [1-4]. The distribution of subjects’ age was similar between netupitant and aprepitant PK studies, and the percentage of female subjects enrolled in the aprepitant study [3] was higher than in the netupitant study [4]. The distribution of subjects’ age and sex was similar between netupitant and aprepitant PD studies [1,2]. Subjects in the 4 studies were either nonsmokers or ex-smokers and did not receive concomitant medications during the study.

All subjects provided written informed consent prior to study participation, and the studies were approved by the local independent ethics committee or institutional review board. The studies were conducted according to the International Council for Harmonisation Good Clinical Practice guidelines, the principles of the Declaration of Helsinki, and local laws and regulations.

PK source data

Mean observed plasma concentration-time curves of netupitant were obtained from a previous randomized, open-label, 2-way crossover, phase 1 study, in which a single oral fixed-dose combination of 300 mg netupitant and 0.5 mg palonosetron was administered to 8 female and 16 male healthy adults (22–45 years old) in both the fed and fasted state; the body mass index ranged from 18.5 to 30 kg/m^2^ (Figure 1 in Calcagnile et al [4]). Blood was sampled predose and at 1, 2, 3, 4, 4.5, 5, 5.5, 6, 8, 12, 24, 48, 72, 96, 120, 144, 192, and 240 h postdose. Arithmetic means of netupitant plasma concentrations at the nominal sampling times from all participants receiving netupitant in the fasted state were used for PK modeling. The mean values were also available from a Helsinn Healthcare SA internal report (data on file). Plasma concentrations of netupitant, netupitant metabolites, and palonosetron were measured using a fully validated liquid chromatography-tandem mass spectrometry (LC-MS/MS) method with stable isotope-labeled internal standards. The lower limit of quantification (LLOQ) of the bioanalytical method was 2 ng/mL for netupitant; the mean precision was <5%, and the accuracy ranged from 98.4% to 107.4% in the selected quality control concentration range, within the accepted limits for validated bioanalytical methods [5]. PK modeling was performed by best fitting a compartmental model to the mean netupitant plasma concentration-time curve observed in healthy adults after oral administration of 300 mg netupitant in a fasted state.

The mean observed plasma concentration-time curves of aprepitant were obtained from an open-label, randomized, 4-period crossover, PK and bioavailability study (Majumdar et al [3]). In part 2 of study 1, an oral capsule of aprepitant 125 mg was administered to 13 female and 12 male healthy subjects (mean age 28 years [range 18–43]) in the fed and fasted state; all subjects were within 20% of their ideal body weight. Blood was sampled predose and at 1, 2, 3, 4, 5, 6, 7, 8, 10, 12, 30, 36, 48, 72, and 96 h after dosing. Aprepitant was measured by LC-MS/MS. The LLOQ of the bioanalytical method was 0.1 ng/mL, the intraday precision was <4.6%, and the accuracy ranged from 98% to 102% of nominal concentrations. PK modeling was performed on the mean aprepitant plasma concentration-time curve observed in healthy adults after oral administration of 125 mg aprepitant in the fasted state. The mean plasma concentrations as a function of the nominal sampling times were obtained from the plot in Figure 2 of the Majumdar et al paper [3], which represents the concentrations on a linear scale, i.e., with no logarithmic transformation of aprepitant concentrations.

PD source data: PET-derived NK_1_ RO

In vivo data on NK_1_ RO by netupitant were obtained from a single-dose, randomized, open‐label, positron emission tomography (PET) study (Figure 3B in Spinelli et al [2]). Six healthy male subjects (mean age 22.3 years [range 20–25]; mean body mass index 23.9 kg/m^2^ [range 21–28 kg/m^2^]) were randomized 1:1:1 to receive 100, 300, or 450 mg of oral netupitant after an overnight fast. [^11^C]‐GR205171, a highly selective, high-affinity NK_1_ receptor antagonist [6,7], was administered as an IV bolus injection at baseline within 1 week before the netupitant dose and at approximately 6, 24, 48, 72, and 96 h after dosing with netupitant. Dynamic brain PET data were acquired in 3D mode over 60 minutes in 17 time frames, within which time [^11^C]‐GR205171 binding could be considered irreversible. Dynamic time‐activity curve data were generated for selected brain regions of interest, including the striatum, and analyzed with a reference Patlak model [8]. The cerebellum was the reference region assumed to have no receptors for specific binding of the tracer [9], and in which the free distribution volume of [^11^C]‐GR205171 was assumed to be the same as in other regions of interest. The percentage of NK_1_ RO was calculated as the relative difference between the net uptake rate of the tracer at baseline and after a single netupitant dose, as estimated from the reference Patlak model.

Data for NK_1_ RO by aprepitant were taken from 2 single-blind, randomized, placebo-controlled PET studies (Table 1 in Bergström et al [1]). In the first study, 12 healthy male subjects (mean age 26 years [range 23–41]; mean weight 75 kg [range 66–92 kg]) were randomized to receive oral aprepitant 10 mg, 30 mg, 100 mg, 300 mg, or placebo 30 minutes after a light breakfast. Subjects were dosed for 14 consecutive days, during which time the brain-to-plasma equilibrium was expected to be relatively independent of the oral dosage. PET imaging was conducted 24 h after the last dose of aprepitant to simulate the trough RO expected with a daily dosing regimen. PET brain images were acquired within 2 weeks before the first aprepitant dose and after an IV bolus of the tracer [^18^F]SPA-RQ, which is a highly selective, high-affinity ligand for the NK_1_ receptor with reversible binding. Total imaging time was 90 minutes with 1- to 6-minute frames, followed by 10-minute frames from 120–160 and 190–240 minutes. In the second study, 4 healthy male subjects (mean 24 years [range 23–25]) were randomized to receive aprepitant 30 mg (n=3) or placebo (n=1) once daily for 14 days. The rest of the PET study was conducted similarly to the first study. PET imaging was conducted 24 h after the last dose of aprepitant, but images were acquired for 87 minutes in 1- to 6-minute frames, followed by 10-minute frames from 120–180, 210–270, and 300–360 minutes. NK_1_ RO was evaluated in the striatum, with the cerebellum as the reference region. The displacement of tracer binding by aprepitant in the striatum was calculated by comparing the pre- and postdose PET scans for each subject according to the linear method [10].

PK modeling

The mean plasma concentration-time curves of netupitant and aprepitant in the source studies were analyzed by compartmental PK modeling following a single oral administration of the agents at therapeutic doses, ie, 300 mg netupitant [4] and 125 mg aprepitant [3]. A 2-compartment model with lag-time and a 1-compartment model with no lag-time best fit the netupitant and aprepitant mean plasma concentration-time curves, respectively, using the Microsoft Excel PKSolver Add-in [11]. Fitting equations are the following:

Two-compartment model for oral administration with lag-time (for netupitant):

Equation 1: Conc = A * e^(-α*(t-tlag))^ + B * e^(-β*(t-tlag))^ – C * e^(-Ka*(t-tlag))^

One-compartment model for oral administration with no lag-time (for aprepitant):

Equation 2: Conc = A * (e^-K10*t^ - e^-Ka*t^)

Where: Conc is the plasma concentration at any time; A, B, and C are macroconstants; α is the distribution rate constant; β is the terminal rate constant; K_a_ is the absorption rate constant; K_10_ is the elimination rate constant from the central compartment 1; t is the time; t_lag_ is lag-time; K_12_ and K_21_ are the transfer rate constants from the central compartment 1 to the peripheral compartment 2, and vice versa. The gut lumen represents the compartment from where netupitant and aprepitant are absorbed. The model Equations 1 and 2 presented above represent the classical equations, respectively, for a two-compartment model with oral dosing and lag-time (for netupitant) and for a one-compartment model with oral dosing with no lag-time (for aprepitant).

Estimated primary model PK parameters were the macroconstants (A, B, C), the absorption rate constant (K_a_), the distribution rate constant (α), the terminal rate constant (β), the elimination rate constant from the central compartment 1 (K_10_), and the lag-time (t_lag_). Secondary PK parameters were the half-lives associated to the absorption (t_1/2,Ka_), distribution (t_1/2,α_), and elimination from central compartment (t_1/2,K10_) phases, the half-life of the terminal phase (t_1/2,β_), the maximum model-predicted concentration (C_max_), the model-predicted peak time (t_max_), the model-predicted area under the plasma concentration-time curve from time zero to infinity (AUC_0-inf_), the C_max_ and AUC_0-inf_ normalized for the administered dose (D) (C_max_/D and AUC_0-inf_/D), the systemic clearance over the bioavailability (F) (CL/F), the inter-compartmental clearance over F (CL_2_/F), the volume of central compartment 1 over F (V/F), and the volume of peripheral compartment 2 over F (V_2_/F). Modeling also estimated also the rates of drug transfer from the central compartment 1 to the peripheral compartment 2 (K_12_), and vice versa (K_21_).

PD modeling

For netupitant, PD model parameters were obtained from the publication by Spinelli et al [2]. A sigmoid maximum drug effect (E_max_) PD model was fitted to the experimental NK_1_ RO% data in the striatum as a function of netupitant plasma concentration data, using the software WinNonlin Professional Edition Version 4.1.b (Pharsight Corporation, Mountain View, CA, USA).

The sigmoid E_max_ model is expressed by Equation 3:

$$RO\%=\frac{E_{max}*{Conc}^{\gamma}}{{EC}_{50}^{\gamma}+{Conc}^{\gamma}}$$

where E_max_ is the maximal occupancy of NK_1_ receptors (NK_1_ RO%), Conc is the plasma concentration of netupitant at the PET scans, EC_50_ is the plasma concentration at which 50% of E_max_ is achieved, and γ is a slope parameter reflecting the shape of the curve.

PD model parameters for aprepitant were estimated by fitting a simple E_max_ model (γ=1) to the NK_1_ RO% data in the striatum after aprepitant administration as a function of aprepitant plasma concentrations using the software Microsoft 365 Excel PKSolver Add-in [11]. The simple E_max_ model is expressed by Equation 4:

$$RO\%=\frac{E_{max}*Conc}{{EC}_{50}+Conc}$$

The NK_1_ RO% and aprepitant plasma concentration data used for aprepitant PD modeling were obtained from the publication by Bergstrom et al (Table 1[1]). The E_max_ model parameters reported in the original publication appeared to lack sufficient accuracy, particularly regarding the E_max_, which was assumed to be 100%, and the EC_50_, which was estimated at approximately 10 ng/mL. Therefore, these parameters were recalculated to allow a correct PK/PD correlation.

Predictions of NK_1_ RO in the striatum

On the basis of the PD model-estimated E_max_, EC_50_, and γ values, and the PK model-predicted plasma concentrations of netupitant and aprepitant at any time (Conc), the NK_1_ RO% profile in the striatum region as a function of time was simulated up to 480 h (20 days) after single oral administration of 300 mg netupitant (the dose bioequivalent to fosnetupitant 235 mg IV), after single oral administration of 165 mg aprepitant (the dose bioequivalent to fosaprepitant 150 mg IV), and after repeated aprepitant dosing with 125 mg on day 1 + 80 mg on days 2–3. RO% predictions for netupitant and aprepitant as a function of time were generated by maintaining constant the PD model estimates of E_max_, EC_50_, and γ parameters, and incorporating the PK model-predicted plasma concentrations of netupitant and aprepitant (Conc) at each time postdose.

Comparisons between netupitant and aprepitant NK_1_ RO were made for days 5, 7, 10, and 20. These time points were selected because day 5 marks the typical end of the assessment period for nausea and vomiting associated with conventional chemotherapy. In addition, a study assessing antiemetic efficacy after day 5 showed fosnetupitant 235 mg IV to be more effective than fosaprepitant 150 mg IV at preventing CINV through day 7 [12]. Finally, nausea following treatment with ADCs like trastuzumab deruxtecan persists primarily through day 10 and, in some cases up to day 20, corresponding to the end of a treatment cycle [13].

| **Supplemental Table 1.** PK and PD properties of orally administered netupitant and aprepitant. | | | |
| --- | --- | --- | --- |
| **Parameter** | **Netupitant (300 mg)** | **Aprepitant (125 mg) day 1 + 80 mg on days 2 and 3** | **Aprepitant (165 mg)** |
| Terminal half-life | 101.6 h | 16.6 h | 16.6 h |
| Time to eliminate 95% of administered dose | ~450 h | ~50 h after third dose  ~100 h after treatment initiation | ~72 h |
| Dose remaining in the body by day 10 | ~20% | ~0.02% | <0.01% |
| Plasma concentration at 240 h postdose | 17.5 ng/mL | 0.34 ng/mL | 0.06 ng/mL |
| E_max_ | 93.0% | 98.1% | 98.1% |
| EC_50_ for NK_1_ RO | 10.2 ng/mL | 10.4 ng/mL | 10.4 ng/mL |
| γ | 1.1 | 1 | 1 |
| RO on day 10 | 59.9% | 3.1% | 0.6% |
| γ, slope parameter reflecting the shape of the curve; E_max_, maximal of NK_1_ RO; EC_50_, effective concentration for 50% NK_1_ RO; NK_1_, neurokinin-1; PD, pharmacodynamic; PK, pharmacokinetic. | | | |

**Supplementary References**

1. Bergström M, Hargreaves RJ, Burns HD, et al (2004) Human positron emission tomography studies of brain neurokinin 1 receptor occupancy by aprepitant. *Biol Psychiatry* 55:1007-1012. https://doi.org/10.1016/j.biopsych.2004.02.007.

2. Spinelli T, Calcagnile S, Giuliano C, et al (2013) Netupitant PET imaging and ADME studies in humans. *J Clin Pharmacol* 54:97-108. https://doi.org/10.1002/jcph.198.

3. Majumdar AK, Howard L, Goldberg MR, et al (2006) Pharmacokinetics of aprepitant after single and multiple oral doses in healthy volunteers. *J Clin Pharmacol* 46:291-300. https://doi.org/10.1177/0091270005283467.

4. Calcagnile S, Lanzarotti C, Gutacker M, Jakob-Rodamer V, Kammerer KP, Timmer W (2015) Evaluation of the effect of food and age on the pharmacokinetics of oral netupitant and palonosetron in healthy subjects: A randomized, open-label, crossover phase 1 study. *Clin Pharmacol Drug Dev* 4:377-386. https://doi.org/10.1002/cpdd.192.

5. US Department of Health and Human Services. Food and Drug Administration. Bioanalytical Method Validation. Guidance for Industry. 2018. https://www.fda.gov/files/drugs/published/Bioanalytical-Method-Validation-Guidance-for-Industry.pdf. Accessed December 9, 2025.

6. Gardner CJ, Armour DR, Beattie DT, et al (1996) GR205171: a novel antagonist with high affinity for the tachykinin NK_1_ receptor, and potent broad-spectrum anti-emetic activity. *Regul Pept* 65:45-53. https://doi.org/10.1016/0167-0115(96)00071-7.

7. Zamuner S, Rabiner EA, Fernandes SA, et al (2012) A pharmacokinetic PET study of NK₁ receptor occupancy. *Eur J Nucl Med Mol Imaging* 39:226-235. https://doi.org/10.1007/s00259-011-1954-2.

8. Patlak CS, Blasberg RG (1985) Graphical evaluation of blood-to-brain transfer constants from multiple-time uptake data. Generalizations. *J Cereb Blood Flow Metab* 5:584-590. https://doi.org/10.1038/jcbfm.1985.87.

9. Caberlotto L, Hurd YL, Murdock P, et al (2003) Neurokinin 1 receptor and relative abundance of the short and long isoforms in the human brain. *Eur J Neurosci* 17:1736-1746. https://doi.org/10.1046/j.1460-9568.2003.02600.x.

10. Wong DF, Gjedde A, Wagner HN (1986) Quantification of neuroreceptors in the living human brain. I. Irreversible binding of ligands. *J Cereb Blood Flow Metab* 6:137-146. https://doi.org/10.1038/jcbfm.1986.27.

11. Zhang Y, Huo M, Zhou J, Xie S (2010) PKSolver: An add-in program for pharmacokinetic and pharmacodynamic data analysis in Microsoft Excel. *Comput Methods Programs Biomed* 99(3): 306-314. https://doi.org/10.1016/j.cmpb.2010.01.007.

12. Hata A, Shiraishi Y, Inui N, et al (2022) Exploratory analysis comparing fosnetupitant versus fosaprepitant for prevention of highly emetogenic chemotherapy-induced nausea and vomiting (CINV): A randomized, double-blind, phase 3 study (CONSOLE). *Oncol Ther* 10:253-262. https://doi.org/10.1007/s40487-022-00188-2.

13. Rugo HS, Bianchini G, Cortes J, Henning JW, Untch M (2022) Optimizing treatment management of trastuzumab deruxtecan in clinical practice of breast cancer. *ESMO Open* 7:100553. https://doi.org/10.1016/j.esmoop.2022.100553.
